# The TARGET cohort study protocol: a prospective primary care cohort study to derive and validate a clinical prediction rule to improve the targeting of antibiotics in children with respiratory tract illnesses

**DOI:** 10.1186/1472-6963-13-322

**Published:** 2013-08-17

**Authors:** Niamh M Redmond, Rachel Davies, Hannah Christensen, Peter S Blair, Andrew M Lovering, John P Leeming, Peter Muir, Barry Vipond, Hannah Thornton, Margaret Fletcher, Brendan Delaney, Paul Little, Matthew Thompson, Tim J Peters, Alastair D Hay

**Affiliations:** 1 Centre for Academic Primary Care, School of Social and Community Based Medicine, NIHR School of Primary Care Research, University of Bristol, Canynge Hall, 39 Whatley Road, Bristol, UK; 2 Research Enterprise and Development, University of Bristol, Senate House, Tyndall Avenue, Bristol, UK; 3 School of Social and Community Based Medicine, University of Bristol, Canynge Hall, 39 Whatley Road, Bristol, UK; 4 Bristol Centre for Antimicrobial Research and Evaluation (BCARE), North Bristol NHS Trust. Southmead Hospital, Bristol, UK; 5 Specialist Virology Centre, Public Health Laboratory Bristol, Public Health England, Myrtle Road, Bristol, UK; 6 Faculty of Health and Social Care, University of the West of England Bristol, Coldharbour Lane, Bristol, UK; 7 Department of Primary Care & Public Health Sciences, King’s College London, School of Medicine, 5th Floor Capital House, 42 Weston Street, London, UK; 8 Department of Primary Care & Population Sciences, University of Southampton, Aldermoor Health Centre, Aldermoor Close, Southampton, UK; 9 Department of Primary Care Health Sciences, Radcliffe Observatory Quarter, Woodstock Road, Oxford, UK; 10 School of Clinical Sciences, University of Bristol, 69 St Michael’s Hill, Bristol, UK

**Keywords:** Cohort study, Children, Respiratory tract infections (RTIs), Primary care, Protocol, Throat swab

## Abstract

**Background:**

Children with respiratory tract infections are the single most frequent patient group to make use of primary care health care resources. The use of antibiotics remains highly prevalent in young children, but can lead to antimicrobial resistance as well as reinforcing the idea that parents should re-consult for similar symptoms. One of the main drivers of indiscriminate antimicrobial use is the lack of evidence for, and therefore uncertainty regarding, which children are at risk of poor outcome. This paper describes the protocol for the TARGET cohort study, which aims to derive and validate a clinical prediction rule to identify children presenting to primary care with respiratory tract infections who are at risk of hospitalisation.

**Methods/design:**

The TARGET cohort study is a large, multicentre prospective observational study aiming to recruit 8,300 children aged ≥3 months and <16 years presenting to primary care with a cough and respiratory tract infection symptoms from 4 study centres (Bristol, London, Oxford and Southampton). Following informed consent, symptoms, signs and demographics will be measured. In around a quarter of children from the Bristol centre, a single sweep, dual bacterial-viral throat swab will be taken and parents asked to complete a symptom diary until the child is completely well or for 28 days, whichever is sooner. A review of medical notes including clinical history, re-consultation and hospitalisations will be undertaken. Multivariable logistic regression will be used to identify the independent clinical predictors of hospitalisation as well as the prognostic significance of upper respiratory tract microbes. The clinical prediction rule will be internally validated using various methods including bootstrapping.

**Discussion:**

The clinical prediction rule for hospitalisation has the potential to help identify a small group of children for hospitalisation and a much larger group where hospitalisation is very unlikely and antibiotic prescribing would be less warranted. This study will also be the largest natural history study to date of children presenting to primary care with acute cough and respiratory tract infections, and will provide important information on symptom duration, re-consultations and the microbiology of the upper respiratory tract.

## Background

Respiratory tract infections (RTIs) in children present a major resource implication for health care services internationally for four reasons. First, they are mostly managed in primary care and are extremely common and costly to service providers, families and employers [1,2]. Second, there is clinical uncertainty in primary care regarding the diagnosis and best management of RTIs, as reflected by the variation in the use of antibiotics in primary care for RTIs between nations [3], general practitioner (GP) practices [4] and clinicians [5,6]. Thirdly, antibiotic prescribing by primary care clinicians in the UK is again on the rise [7] and finally combined with the slowing in the development of new antibiotics, the overuse and misuse of existing antibiotics is associated with the development and proliferation of antimicrobial resistance between [8] and within [9] nations as well as individuals [8-10]. The use of antibiotics also leads to the subsequent ‘medicalisation of illness’ in which patients believe they should consult for similar symptoms in the future [11]. Thus, the increasing or decreasing use of antibiotics can lead to vicious or virtuous re-consultation cycles.

A number of key publications have highlighted the need for more research to define the appropriate use of antibiotics and health care resources for RTIs, if the public health disaster of ineffective antibiotics for serious infections is to be averted [12-15]. One particular focus was the requirement to establish which clinical features of children presenting in primary care with RTIs are associated with the development of serious complications and the need for hospitalization [16]. A prognostic tool identifying the risk of hospitalisation could be a key driver to rationalising antibiotic prescribing [17].

Clinical prediction rules are designed to reduce clinical uncertainty in an outcome (such as a child’s risk of hospitalisation) by assessing the strength of association between the risk of it occurring and baseline characteristics (for example, socio-demographic characteristics or symptoms and signs of illness). Further insight might be gained into the variability of the prognosis by measuring bacterial and viral aetiology. For example, co-infections of viral and bacterial pathogens may be associated with poor prognosis [18-22]. To our knowledge, no study has characterised the bacterial and viral flora of a representative sample of children presenting with RTI to primary care, and investigated their prognostic significance.

The aim of the TARGET cohort study is to establish the clinical (signs and symptoms) and microbiological (viral and bacterial pathogens) factors influencing the prognosis of children presenting to primary care with acute cough and RTI. This study is part of a wider programme of research to improve the care of children with RTI including: (i) systematic reviews regarding the prognosis of RTI in children and interventions to reduce antibiotic use [23,24] and (ii) qualitative research into the consultation experiences of parents and clinicians [25]. The final component of the programme is to test the clinical and cost-effectiveness of a fully developed clinical prediction rule based within a multi-faceted intervention for children presenting to primary care with acute RTI.

## Methods/design

The TARGET cohort study is a multi-centre, prospective cohort study to derive and validate a clinical rule using symptoms, signs, demographic and microbiological factors to predict complications of acute cough in children presenting to primary care with RTI. The study is aiming to recruit 8,300 children from primary care sites attached to four study centres (Bristol, London, Oxford and Southampton, UK) between July 2011 and end of June 2013. The Bristol study centre also collects single sweep dual viral and bacterial throat swabs from recruited children and asks parents/carers to complete a symptom and medication diary for up to 28 days after recruitment. The study was approved by the South West Central Bristol Research Ethics Committee, UK (reference number: 10/H0102/54) and research governance approvals obtained across all areas prior to the start of recruitment in those areas.

### Participant eligibility

**Figure 1:**
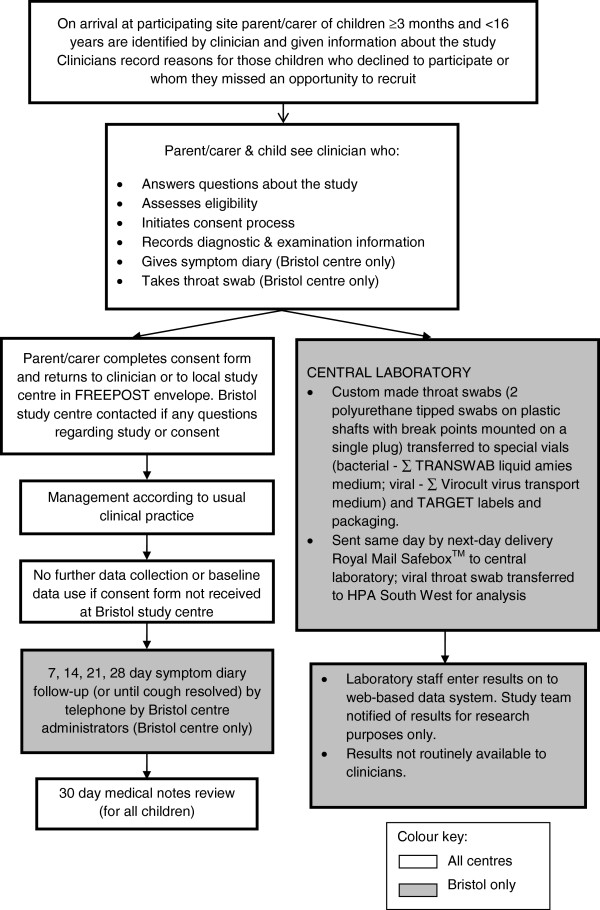
TARGET cohort study recruitment process flowchart.

Children are included if they are: ≥3 months to <16 years; present with a RTI with cough of ≤28 days duration prior to consultation; presenting with illnesses such as asthma, epilepsy or diabetes and RTI, including infective exacerbation of asthma as well as children who require same day hospital assessment or admission. Children are excluded if they present with acute, non-infective exacerbation of asthma; they present with RTI without cough or cough of >28 days; they are considered to have a high risk of serious infection (for example, immunocompromised, cystic fibrosis, splenectomy); they require a throat swab for the clinical care of the child (Bristol centre only – see later for explanation); parents/carers/children have temporarily registered with the National Health Service (NHS) primary care site (GP practice, Walk-in centres, GP Out of Hours centres or polyclinics) and are likely to be unregistered/non-resident within a month; parent/carers/children are unable or unwilling to assist with the study; they are already recruited to the TARGET cohort study or involved in other research or have recently (within 28 days) been involved in similar clinical research. Figure 1 details the recruitment process for participants.

The primary outcome, to be collected by a medical notes review of all children, is any hospital admission for lower RTI (for instance, bronchiolitis, pneumonia or empyema) in the 30 days following recruitment. Secondary outcomes, collected in children recruited at the Bristol centre using a parent completed symptom diary and medical notes review are: referral from GP/Out of Hours to secondary care; symptom duration and severity; re-consultations; antibiotic prescribing and antibiotic consumption in the 30 days following recruitment.

### Clinician and participant recruitment

First, general practices express an interest in participating in the TARGET cohort study via the UK primary care research network (PCRN). Once identified, practices and prescribing clinicians (GPs or prescribing nurses) are invited to sign up and complete agreement forms. Information regarding the total patient list size as well as the number of children in the eligibility age range are obtained. By agreement with the study centre, individual sites are able to organise recruitment to suit the needs of the organisation; for example, sites can enlist the support of their practice nurses to help with recruitment.

Only clinicians who self-report use of antibiotics in 30% or fewer children with RTIs are invited to recruit. This criterion was set to reduce the effect of what is known as ‘confounding by indication’. This is the phenomenon whereby the way primary care clinicians already decide to which children they will prescribe antibiotics is to an extent associated with any clinical prediction tool that might be developed. This plausible possibility will of course in itself alter the risk of the hospitalisation outcome amongst children in the study, and hence in turn reduce the apparent associations between some risk factors and the outcome. In addition, in order to ensure an unbiased sample of the full illness spectrum, clinicians are asked to identify a systematic recruitment strategy. Clinicians are asked to specify how many children per week they expect to recruit. To measure the success of clinicians’ recruitment strategies, and to establish the generalisability of recruited children to the population as a whole, clinicians are asked to complete some brief details on those children that they invited to participate but declined and those they missed. Clinicians are asked to use the online database or complete and send postcards to record the child’s illness severity and the reason for decline or missed opportunity to recruit. A ‘missed’ opportunity to recruit is defined as those children who would have normally met the clinician’s own strategy criteria and the study inclusion criteria but where the clinician decided not to recruit for whatever reason (for instance, the clinician forgot or was too busy).

All clinicians are trained in the study processes of obtaining consent, assent (for children aged 11 and over) and completing the baseline data collection form known as the case report form (CRF). For the clinicians recruiting children to the Bristol study centre, clinicians are additionally trained in how to take a ‘single sweep’ dual bacterial-viral TARGET specific throat swab. A video (see http://www.youtube.com/watch?v=eiM0arJGYT0&feature=youtu.be) was created to illustrate and standardise the taking and packaging of the throat swab to send to the local laboratory for processing. After training in the study recruitment processes, clinicians are given participant study packs, vial stands, practice and clinician instruction folders and clinician specific logon details for the online study database and are able to commence recruitment.

### Data collection

#### Baseline case report form

Once informed consent (and assent for children aged 11 or over) has been obtained, recruiting clinicians complete a CRF (see Additional file 1) for each child to record socio-demographic information, carer-reported symptoms (including duration and severity of symptoms in the past 24 hours), clinician observed signs and clinical management data, and treatment (such as no, delayed or immediate antibiotic prescription).

### Microbiological data (Bristol study centre children only)

#### Throat swab design and transport

Bristol centre research staff in collaboration with the throat swab manufacturer, Medical Wire Ltd (Wiltshire, UK) designed a kit consisting of a dual bacterial-viral throat swab suitable for use in small children, requiring one sweep either side of the child’s throat.

The swab kit contains the two swabs (two polyurethane foam tipped swabs on plastic shafts with break points, mounted on a single plug (swab tip 15 mm, shaft 110 mm, handle 30 mm) and clear plastic 13 mm diameter 84 mm long tubes, one with a purple cap for bacteriology testing (containing ∑ TRANSWAB liquid Amies medium) and one with a green cap for virology testing (containing ∑ Virocult virus transport medium and 3 plastic beads). The kit is provided in a sterile peel pouch.

Once the recruiting clinician has taken the throat swab, swabs are individually broken off into the vials in line with standardised instructions. Throat swabs are sent, using a next-day delivery Post Office Safebox™ to the Bristol Centre for Antimicrobial Research and Evaluation (BCARE) at Southmead Hospital, Bristol, UK (bacteriology) from where the virology swab is forwarded to the Specialist Virology Centre at the South West Health Protection Agency (HPA) Laboratory, Bristol UK (virology).

### Processing of bacterial swabs

The bacteriological swab is vortex mixed and the transport medium is innoculated onto agar plates using both standard streaking method and spiral plating. Following overnight incubation of the agar plates, colonies morphologically consistent with *Streptococcus pneumoniae*, *Haemophilus influenzae*, *Moraxella catarrhalis*, beta haemolytic streptococci (A, C, F, G), or *Staphylocococcus aureus* are identified by standard laboratory tests. For each sample, the presence or absence of these target organisms is recorded along with a semi-quantitative colony count (from the streak plates) and a quantitative colony count (from the spiral plates). Total aerobic bacterial count is also recorded.

### Processing of viral swabs

On receipt of the virology swab sample, the virology lab staff test the sample for common respiratory viruses using real-time polymerase chain reaction (PCR) assays that have been extensively validated and quality assured for routine viral diagnosis. A 0.1 mL aliquot of transport medium from each swab sample is processed to recover microbial nucleic acids using a robotic nucleic acid extraction method. Real-time PCR assays are used to detect the presence or absence of the following viruses and bacteria: influenza A and B, respiratory syncytial viruses, metapneumoviruses, parainfluenzavirus types 1–4, adenovirus, rhinovirus, enterovirus, parechovirus, coronavirus, bocavirus, *Bordetella pertussis*, *Bordetella parapertussis*, *Mycoplasma pneumoniae* and *Chlamydia pneumoniae*.

### Results of throat swabs

The microbial results of the samples are not made available to the recruiting clinicians because this could influence the subsequent care of the child (this is also the reason for excluding children where the clinician feels a throat swab is necessary for clinical management). Additionally, throat swabs are rarely taken in routine clinical care for children with cough and the clinical significance of the results is not established.

### Symptom diary (Bristol study centre children only)

Parents/carers are asked to complete a symptom diary, based on a previously validated study diary [26], either on paper or online until symptom resolution or day 28, whichever is soonest. This is supported by weekly telephone contact, similar to that successfully used to obtain >85% follow up data in previous studies [27,28], to record symptom duration, severity and medicine consumption. Parents/carers are also invited to answer some simple background information questions to establish whether the study is representative of the UK population as a whole. Standardised study procedures are used to manage, implement and record the outcomes of the daily telephone calls to support recruited families in completing the diary. Several processes and strategies (including voicemail messages, short message service (SMS) text messages and postcard reminders) are utilised to maximise diary completion and return.

### Primary care notes review

#### 30 day primary care notes review

The primary care records of all children are examined for hospital admissions and primary care re-consultations in the 30 days following recruitment. Information on prior immunisations and antibiotics, are also collected from the primary care record. Primary care notes reviews commence at least 3 months after the date the child was recruited, in order to allow hospital discharge letters to reach the primary care notes. Double notes reviewing is undertaken for 1% of the participants to estimate inter-reviewer error.

### Secondary care notes review

Since primary care discharge summaries do not always contain full details of hospitalisations, for the anticipated 160 children (40 per centre) identified with a hospital admission, secondary care records will be examined for lower RTIs (such as ‘chest infection’ , bronchitis, bronchiolitis, pneumonia, lung abscess or empyema) requiring interventions available only in hospital (including oxygen, intravenous fluids or intravenous antibiotics).

### Data management systems

A large study such as this requires highly efficient data management systems. All data for the study are collected on either web-based or paper forms. The web-based data collection and management system was created by the Bristol and London teams, with input from all centres and collaborators. As a time-saving facility, when a recruiting clinician completes the CRF as an online form, a summary of the data collected is created for the clinician to copy and paste into the recruited child’s medical record. The symptom diary is available for Bristol centre parents to complete online. Parents/carers access their child’s online symptom diary by entering the child’s study identification number and date of birth. Only parents/carers who have completed a valid consent form can use this facility. Each study centre has an MS Access (Microsoft, US) database, which facilitates primary care site, recruiting clinician, and notes review management. The Bristol study centre holds a combined version, updated at least weekly, so that weekly recruitment and notes review completion monitoring summaries are produced and reviewed.

### Service user involvement

Parents have been consulted throughout the whole process of the study to inform design, conduct and data collection, including preparation of the study paperwork and marketing materials for the recruitment phase, feasibility of the dual throat swabs and design and use of the symptom diary on paper and online. 13 different independent parents gave feedback on a variety of different aspects of the study and at least 20 different children of various ages provided feedback on study aspects involving children (such as the child information sheet and study stickers).

### Study analysis

#### Sample size justification

With a 5% two-sided significance level and 80% power, assuming a hospitalisation rate of 2% [29,30] and an individual clinical characteristic prevalence of 25% [29], a sample of 2,588 will allow detection of an odds ratio (OR) of 2.4 of associated signs and symptoms (ORs of between 2.5 and 5 have been observed previously [29,31]). If hospitalisation rates are lower at 1% and symptoms less prevalent at 10%, a sample of 2,390 will allow detection of an odds ratio of 4.5 for associated signs and symptoms (assuming the outcome is hospitalisation and the exposure is the presence of a given clinical characteristic). Taking account of the need to collect derivation and validation data (and dividing the dataset in a 50:50 split, an antibiotic prescribing rate of 30% (these children will not be included in the analyses due to the potential for confounding by indication) and 10% attrition, a total sample of 8,216 will be required. This sample size will also give more scope to look at several variables (maximum one variable for every 10 hospitalised cases) [32] in the multivariable regression model.

With a 5% two-sided significance level and 80% power, assuming a mixed viral and bacterial infection rate of 10% [33], a sample of 1,610 children will be needed to detect the 10% of children with the most prolonged/severest symptoms with an OR of 2. Allowing for symptom diary and microbiological data loss in 15%, 1,894 children are required to be recruited from the Bristol centre (sub-set of children).

### Statistical analysis

The main statistical analyses will be carried out according to the study analysis plan. First, descriptive statistics will be used for: children’s clinical and microbiological characteristics; hospitalisations; symptom duration and severity; and primary care re-consultations; immunisation and antibiotic exposures pre-recruitment; antibiotic prescribing and consumption. For outcomes such as hospitalisation, re-consultation, lengthy symptom duration and antibiotic prescribing testing will include chi-square for categorical variables (or Fisher’s exact test when the expected cell is less than 5) and both a parametric and non-parametric approach for continuous variables depending on the nature of the distribution. Internal validation will be used for assessing the value of a derived rule [34]. We will also explore alternative approaches including bootstrapping and subdivision of the sample on a selective rather than a random basis so as to assess the robustness of the rule in different circumstances (such as geographical location). The clinical prediction rule will be developed based on the linear predictor in a logistic regression model in which the outcome variable is hospitalisation among children not treated with antibiotics. Candidate prognostic variables will be categorised into demographic background, and symptoms and signs (for example, overall illness severity, fever, shortness of breath). Variables included in logistic regression models will be based on an ‘inclusive’ p-value threshold of 0.1 in the univariable analysis. Significance in the multivariable model will take into account the number of variables tested using multiple testing methods such as the Bonferroni Correction. We will check for nonlinear effects of continuous variables, and will examine candidate interactions specified *a priori*. Such effects will be included in the final models as necessary. We will begin by examining the predictive value (based on diagnostic odds ratios and confidence interval (CI) statistics) of the best predictors from the socio-demographic variables. We will then examine the additional prognostic value of presenting signs and symptoms (compared with socio-demographic data alone).

Diagnostic and prognostic models that are developed using p-value-based variable selection will inevitably suffer from statistical over-optimism. Therefore, the final models will be validated using the second dataset, and the published decision rule will be based on the linear predictors from the model re-estimated in this validation dataset. In the final stages of analysis, we will examine the sensitivity and specificity of the linear predictor, based on a set of chosen thresholds for positivity. A comparison will be made between the results obtained from the validation and the use of shrinkage based approaches applied to the original development dataset [35]. The final clinical rule will be characterised based on its sensitivity and specificity, and positive and negative predictive values.

Univariable and multivariable logistic regression models will also be used to investigate the unadjusted and adjusted strengths of association between the presence/absence of co-viral/bacterial carriage and prolonged symptoms. Model entry will be set with a threshold p-value of 0.1, a clinically informed (rather than automated stepwise) selection procedure will be used and the potential interaction of predictor variables, in particular any interaction between specific symptoms, will be tested. In addition, we will explore the effects of the semi-quantitative bacteriology (scanty, moderate and heavy growth of target organisms) and virology (real time PCR cycle threshold of detection (C*t* value)) on symptom duration and we will investigate interactions between the bacteriology and antibiotic treatment.

## Discussion

The TARGET study is, to our knowledge, the first study primarily designed to improve the targeting of antibiotics via the development of a clinical prediction rule for appropriate antibiotic prescribing in children presenting to primary care with acute cough. This study addresses repeated calls from policymakers and the research community to appropriately target the use of resources in RTIs, and attempts to address the ‘ticking timebomb’ of antibiotic resistance [12-15]. As one of the most frequent users of primary care, there is a need for more appropriate, evidence-based antibiotic prescribing to reduce unnecessary antimicrobial resistance and to reduce the expectation of parents to consult and receive antibiotics for cough.

The main strengths of the TARGET study arise from the large scale of the design. We aim to recruit at least 8,300 children across 4 study centres, thereby maximising the potential for generalisability. This study will be the largest natural history study to date of children presenting to primary care with acute cough and RTI, providing important data on symptoms and signs presented in primary care, re-consultations and hospitalised cases. In a subset of children, symptom severity, duration and a broad panel of potential respiratory pathogens of the upper respiratory tract will be made available. The main challenge in this study will be the ambitious recruitment targets necessitated by the rarity of the outcome; hospitalisation is an infrequent event in RTIs occurring in 1-2% of those presenting to primary care [29].

The potential for a tool to help clinicians to decide whether a child presenting with RTI requires hospitalisation is highly desirable although relatively speaking this is a rare event in the day-to-day practice of an individual clinician. Perhaps of more benefit is a tool that can distinguish a much larger population of children that not only do not need to be hospitalised but could avoid antibiotics. A clinical prediction tool could potentially deliver both and is thus worth the effort of such a large and complex study.

In summary, the TARGET study will be one of the largest studies of its kind undertaken in primary care. The anticipated prediction rule aims to improve the targeting of antibiotics for those children who are most likely to benefit, whilst also identifying those children where treatment is unlikely to be required. This will provide much needed guidance for the frequently encountered conundrum of appropriate management of children presenting to primary care with RTIs.

## Abbreviations

RTIs: Respiratory tract infections; GP: General practitioner; NHS: National health service; PCRN: Primary care research network; CRF: Case report form; BCARE: Bristol centre for antimicrobial research and evaluation; HPA: Health protection agency; PCR: Polymerase chain reaction; SMS: Short message service; OR: Odds ratio; CI: Confidence interval; Ct: ValueCycle threshold value.

## Competing interests

The authors declare that they have no competing interests.

## Authors’ contributions

PSB, BD, MF, ADH, AML, JPL, PL, PM, MT, and TJP are responsible for developing the research question. PSB, HC, BD, RD, MF, ADH, AML, JPL, PL, PM, NMR, HT, MT, TJP and BV are responsible for the study design and collection of data. NMR, RD and HC are responsible for study management and co-ordination. NMR drafted the paper. All authors read and approved the final manuscript.

## Pre-publication history

The pre-publication history for this paper can be accessed here:

http://www.biomedcentral.com/1472-6963/13/322/prepub

## Supplementary Material

Additional file 1 The TARGET cohort study case report form (CRF). Click here for file

## UK clinical research network (UKCRN) portfolio registration

The cohort study is registered on UK Clinical Research Network Portfolio as ‘The TARGET study’ reference number 9334.
