# Supplementary material for: The TARGET cohort study protocol: a prospective primary care cohort study to derive and validate a clinical prediction rule to improve the targeting of antibiotics in children with respiratory tract illnesses
Source: BMC Health Serv Res. 2013 Aug 17;13:322. doi: 10.1186/1472-6963-13-322 (PMC3765099; doi:10.1186/1472-6963-13-322)
Supplement: Additional file 1 — The TARGET cohort study case report form (CRF). [file 1472-6963-13-322-S1.pdf]

CASE REPORT FORM

TARGET

ID 999999

Today's date

D D / M M / 2 0 Y Y

Informed consent for study obtained ☐$_1$

Background information

DOB D D / M M / Y Y Y Y

Gender ☐ Female$_0$ ☐ Male$_1$

Ethnicity ☐☐

PTO for codes, if other ethnicity, please describe below

☐☐☐☐☐☐☐☐☐☐☐☐☐

Mother's age ☐☐

children in home (inc. unwell child) ☐☐

Does the mother smoke? ☐ No$_0$ ☐ Yes$_1$ ☐ Don't know$_{88}$

Mother still breast feeding child at 3 months? ☐ No$_0$ ☐ Yes$_1$ ☐ Don't know$_{88}$

Carer reported symptoms

How unwell does the **parent** consider the child to be?

Well ☐0 ☐1 ☐2 ☐3 ☐4 ☐5 ☐6 ☐7 ☐8 ☐9 ☐10 Very unwell

Duration of illness ☐☐ days

Has illness got a lot worse recently? ☐ No$_0$ ☐ Yes$_1$

If Yes, how many days ago did it start to get worse? ☐☐ days

Symptoms present	During illness? No$_0$	During illness? Yes$_1$	*If yes*	Last 24 hours? No$_0$	Last 24 hours? Yes$_1$	*If yes*	*Severity in last 24 hours (tick **one**)* Mild$_1$	Moderate$_2$	Severe$_3$
Dry cough	☐	☐	>	☐	☐	>	☐	☐	☐
Productive/ wet cough	☐	☐	>	☐	☐	>	☐	☐	☐
Barking/ croupy cough	☐	☐	>	☐	☐	>	☐	☐	☐
Blocked or runny nose	☐	☐	>	☐	☐	>	☐	☐	☐
Change in cry	☐	☐	>	☐	☐	>	☐	☐	☐
Breathing faster than normal (shortness of breath)	☐	☐	>	☐	☐	>	☐	☐	☐
Wheeze or whistling in the chest	☐	☐	>	☐	☐	>	☐	☐	☐
Fever	☐	☐	>	☐	☐	>	☐	☐	☐
Chills/ shivering	☐	☐	>	☐	☐	>	☐	☐	☐
Diarrhoea	☐	☐	>	☐	☐	>	☐	☐	☐
Vomiting (including after cough)	☐	☐	>	☐	☐	>	☐	☐	☐
Taking fewer fluids/ milk feeds	☐	☐	>	☐	☐	>	☐	☐	☐
Eating less	☐	☐	>	☐	☐	>	☐	☐	☐
Low energy/ fatigue/ lethargy	☐	☐	>	☐	☐	>	☐	☐	☐
Disturbed sleep	☐	☐	>	☐	☐	>	☐	☐	☐
Passing urine less often/ dryer nappies	☐	☐	>	☐	☐	>	☐	☐	☐
Please tick NA if the child is too young/ uncommunicative for the parent to know about the following > NA ☐$_1$									
Chest/ shoulder pain	☐	☐	>	☐	☐	>	☐	☐	☐
Headache	☐	☐	>	☐	☐	>	☐	☐	☐
Muscle aches all over	☐	☐	>	☐	☐	>	☐	☐	☐
Confusion/ disorientation	☐	☐	>	☐	☐	>	☐	☐	☐

Clinician examination and management

	Absent$_0$	Present$_1$
Pallor	☐	☐
Grunting	☐	☐
Nasal flaring	☐	☐
Stridor	☐	☐
Inter/ subcostal recession	☐	☐
Inflamed pharynx/ tonsils	☐	☐

	Absent$_0$	Unilateral$_1$	Bilateral$_2$
Wheeze	☐	☐	☐
Crackles/ crepitations	☐	☐	☐
Bronchial breathing	☐	☐	☐

Temperature ☐☐.☐ ºC

Pulse ☐☐☐ bpm

Respiratory rate ☐☐ bpm

O_2 sat ☐☐☐ % ☐ Unable to take O_2 sat/no equipment

Consciousness level ☐ normal$_0$ ☐ irritable$_1$ ☐ drowsy$_2$

Capillary refill time ☐ 2 seconds or less$_0$ ☐ 3 seconds or more$_1$

How unwell do **you** consider the child to be?

Well ☐0 ☐1 ☐2 ☐3 ☐4 ☐5 ☐6 ☐7 ☐8 ☐9 ☐10 Very unwell

Throat swab taken? ☐ No$_0$ ☐ Yes$_1$

If **No**, reason:

☐ Child refusal$_1$

☐ Parent refusal$_2$

☐ Other (specify)$_3$

☐☐☐☐☐☐☐☐☐☐☐☐

☐☐☐☐☐☐☐☐☐☐

Main working respiratory tract diagnosis ☐☐☐☐☐☐☐☐☐☐☐☐☐☐

My gut feeling is 'something is wrong' ☐ No$_0$ ☐ Yes$_1$

Antibiotics prescribed? ☐ No$_0$ ☐ Yes, immediate$_1$ ☐ Yes, delayed$_2$ by ☐☐ days

Referral for acute admission today? ☐ No$_0$ ☐ Yes$_1$

Ethnicity showcard

To which of these ethnic groups do you consider your child belongs? / What is your ethnic group?

White

1 British

2 Irish

3 Any Other White background (please describe to the doctor or nurse)

Mixed

4 White and Black Caribbean

5 White and Black African

6 White and Asian

7 Any Other Mixed background (please describe to the doctor or nurse)

Asian or Asian British

8 Indian

9 Pakistani

10 Bangladeshi

11 Any Other Asian background (please describe to the doctor or nurse)

Black or Black British

12 Caribbean

13 African

14 Any Other Black background (please describe to the doctor or nurse)

Chinese

15 Chinese

Other ethnic group

16 Any Other (please describe to the doctor or nurse)

To the clinician: please record the number corresponding to the patients response in the case report form ethnicity box along with any additional description about their ethnicity the patient may wish to provide

For TARGET study office use only

Date received at Bristol centre
